# Urine Aquaporin-2: A Promising Marker of Response to the Arginine Vasopressin Type-2 Antagonist, Tolvaptan in Patients with Congestive Heart Failure

**DOI:** 10.3390/ijms17010105

**Published:** 2016-01-14

**Authors:** Teruhiko Imamura, Koichiro Kinugawa

**Affiliations:** 1 Department of Therapeutic Strategy for Heart Failure, Graduate School of Medicine, University of Tokyo, Tokyo 113-8655, Japan; te.imamu@gmail.com; 2 Second Department of Internal Medicine, University of Toyama, Toyama 930-0194, Japan

**Keywords:** heart failure, congestion, chronic kidney disease, hyponatremia

## Abstract

Aquaporin-2, a member of the aquaporin family, is an arginine vasopressin-regulated water channel expressed in the renal collecting duct, and a promising marker of the concentrating and diluting ability of the kidney. The arginine vasopressin type-2 antagonist, tolvaptan, is a new-generation diuretic; it is especially indicated in patients with decompensated heart failure refractory to conventional diuretics. However, the ideal responders to tolvaptan have not yet been identified, and non-responders experience worse clinical courses despite treatment with tolvaptan. Urine aquaporin-2 has recently been demonstrated as a promising predictor of response to tolvaptan. We here validated aquaporin-2-guided tolvaptan therapy in patients with decompensated heart failure. Long-term efficacy of tolvaptan treatment in the responders defined by aquaporin-2 needs to be validated in the future prospective study.

## 1. Aquaporin-2 in Patients with Congestive Heart Failure (HF)

The aquaporin family has various activities in the human body, such as mediating water transport, and cell adhesion, migration, proliferation, and differentiation [1]. Of the aquaporin family members, aquaporin-2 has been characterized as an arginine vasopressin (AVP)-regulated water channel protein translocating between the apical plasma membrane and subapical vesicles in the principal cells of the collecting duct [2].

**Figure 1:**
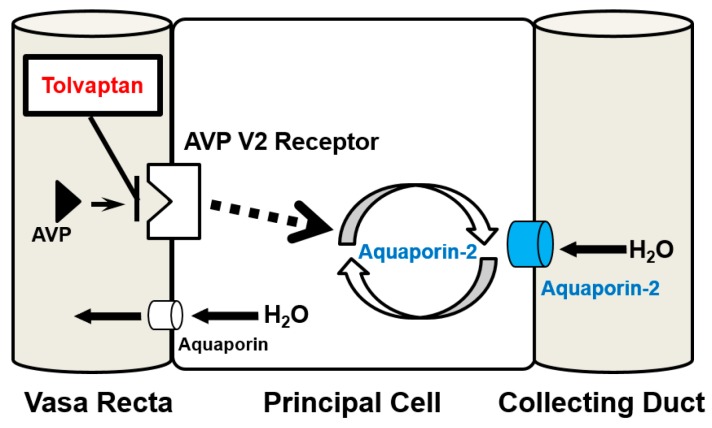
The relationship between arginine vasopressin, aquaporin-2, and tolvaptan in the collecting duct. AVP, arginine vasopressin.

The mechanisms of aquaporin-2-mediated water retention have been studied for the past 20 years [3]. Secreted AVP binds to the AVP type-2 (V_2_) receptor located at the basolateral membrane of principal cells (Figure 1). Activation of the V_2_ receptor triggers the trafficking of aquaporin-2 from intracellular storage vesicles to the apical membrane by cAMP-dependent phosphorylation of aquaporin-2 [4,5]. Translocation of aquaporin-2 to the apical membrane increases water permeability, thereby increasing urine osmolality (U-OSM) [6].

Approximately 3% of aquaporin-2, in particular phosphorylated and translocated aquaporin-2, in the kidney tissue is excreted in urine [7]. Therefore, urine aquaporin-2 is considered a marker of collecting duct responsiveness to AVP [8].

Several techniques have been developed to quantify urine aquaporin-2, such as radioimmunoassay, Western blotting, and sandwich enzyme-linked immunosorbent assay [7,9,10], and urine aquaporin-2 has been quantified in various clinical conditions including pregnancy [11], liver cirrhosis [12], syndrome of inappropriate secretion of antidiuretic hormone [5], diabetes insipidus [4], and HF [13,14].

In patients with congestive HF, AVP is inappropriately secreted through the activation of a non-osmotic pathway owing to reduced effective circulating volume, despite peripheral water retention [15,16,17,18]. Secreted AVP stimulates the expression and translocation of aquaporin-2 at the apical membrane of the collecting duct [4,5], thereby facilitating water retention and successive hypervolemic hyponatremia (Figure 1) [15]. Therefore, urine aquaporin-2 level increases in patients with HF owing to inappropriately elevated serum AVP level. Plasma level of AVP increases in association with HF progression, accompanied by activation of the renin-angiotensin-aldosterone system and sympathetic nerve system, which facilitate water retention [15,16,17,19]. Many studies have demonstrated that hyponatremia is a predictor of morbidity and mortality in patients with HF [20,21,22,23].

## 2. Tolvaptan and Its Responsiveness

Tolvaptan, the only commercially available vasopressin V_2_ receptor antagonist, thus far, has been recently developed as a promising agent to treat patients with congestive HF refractory to conventional diuretics [24]. It has a unique therapeutic target: it blocks AVP-aquaporin-2 pathway, and increases the excretion of electrolyte-free water in urine (Figure 1). Many studies have demonstrated the short-term efficacy and safety of tolvaptan; it also ameliorates symptomatic congestion, normalizes hyponatremia to maintain hemodynamics and the sparing renal function, and terminates the vicious cycle of HF described above [25,26,27,28,29,30,31,32,33,34,35]. However, these benefits may not be expected in non-responders, whose urine volume does not increase despite the administration of tolvaptan [36]. Tolvaptan could not normalize serum sodium concentration, ameliorate symptomatic congestion, and improve renal function in non-responders. Moreover, indeterminate administration of tolvaptan to non-responders may result in a loss of the optimal timing of intensive treatment [37].

Tolvaptan was not found to be superior to placebo in terms of long-term survival rate in the Efficacy of Vasopressin Antagonist in HF Outcome Study with Tolvaptan (EVEREST) trial [38]. Considering that tolvaptan demonstrated improved survival rate over the placebo in patients with hyponatremia in this subanalysis [39], it may improve long-term prognosis only in a specific population. Therefore, it is necessary to identify the optimal population, *i.e.*, responders [40,41].

## 3. Tolvaptan and Aquaporin-2

Several studies recently demonstrated that unresponsiveness to tolvaptan is associated with decreased renal function [42,43,44]. Older patients with chronic kidney disease have a trend of physiological insipidus mellitus, and have reduced urine concentrating ability. Tolvaptan may not be able to inhibit the already-extinct AVP-aquaporin-2 system in the collecting duct, and the residual function of the collecting duct is essential for response to tolvaptan [40,45]. Therefore, urine aquaporin-2 has been evaluated as a predictive “marker” of responsiveness to tolvaptan [46].

We recently demonstrated that higher urine aquaporin-2 level at baseline relative to plasma AVP was a novel predictor of responsiveness to tolvaptan [37], because urine aquaporin-2 level increases in association with AVP stimulation under the well-preserved function of the collecting duct. Higher level of urine aquaporin-2 is associated with elevated U-OSM in the responders [37]. Thus, U-OSM could be an alternative to urine aquaporin-2 for the prediction of responses to tolvaptan [40].

In contrast, urine aquaporin-2 levels in non-responders were not detectable, regardless of serum AVP level [37]. In general, expression level of aquaporin-2 decreases in patients with chronic kidney disease [47]. Sato *et al.* showed that aquaporin-2 is not expressed in the renal tissue of non-responders with diabetes nephropathy [48]. However, not all patients with chronic kidney disease have reduced urine excretion of aquaporin-2. Some patients have preserved function of the collecting duct despite decreased glomerular filtration ratio, and such patients are classified as responders to tolvaptan [37]. However, several studies reported that even patients with advanced chronic kidney disease responded to tolvaptan [43,44,45,49,50]. Therefore, urine aquaporin-2 could be a novel predictor to estimate the responders to tolvaptan especially among those with chronic kidney disease.

**Figure 2:**
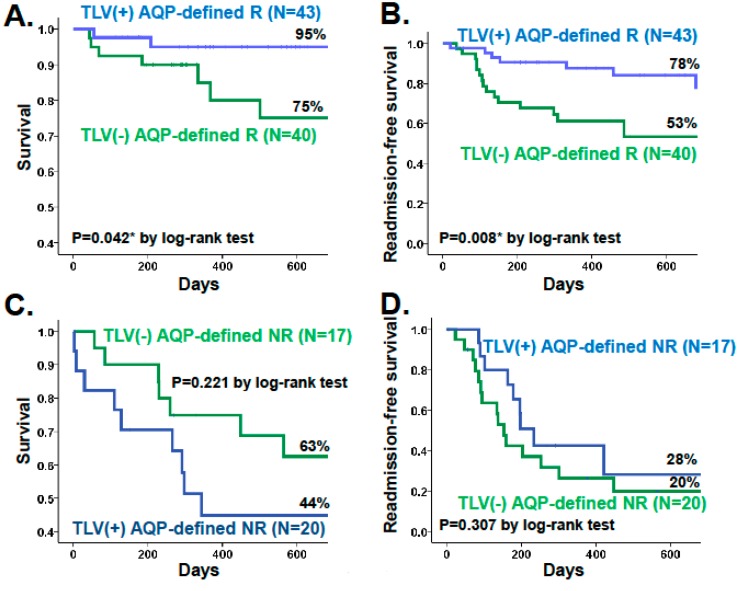
Kaplan-Meier curves showing survival and readmission-free survival rate stratified by the administration of tolvaptan in responders (**A**,**B**) and non-responders (**C**,**D**). R, responder; NR, non-responder; TLV, tolvaptan, AQP, aquaporin.

Long-term improvements in survival and re-admission-free survival rate were observed in the responders receiving tolvaptan, who were defined by urine aquaporin-2, although the study was retrospective (Figure 2A,B) [37]. In this study, potential response was defined as baseline urine aquaporin-2/serum AVP level >1.4 × 10^3^ L/gCre, and those receiving tolvaptan were compared with propensity score-matched control group. In contrast, non-responders, whose urine aquaporin-2/erum AVP level <1.4 × 10^3^ L/gCre, could neither achieve improvement in survival nor in re-admission-free survival rate despite tolvaptan administration, compared with propensity score-matched non-responders without tolvaptan (Figure 2A,B). Tolvaptan ameliorated symptomatic congestion, normalized hyponatremia, improved renal function, and reduced the dose of diuretics in the aquaporin-defined responders. These effects may improve patients’ prognosis during long-term tolvaptan treatment. Prospective randomized trials are warranted to evaluate long-term improvement in the prognosis of the aquaporin-defined responders receiving tolvaptan.

Measurement of urine aquaporin-2 is also useful in confirming the response to tolvaptan in a timely fashion. Martin *et al.* showed that urine aquaporin-2 level decreased accompanied by an increase in urine volume from 2 h after the administration of tolvaptan [14]. Udelson *et al.* demonstrated a decrease in urine osmolality at 4 h after tolvaptan administration, which indicates decreased aquaporin-2 activity [31]. The decreased level of urine aquaporin-2 is restored the next morning, but does not reach the baseline level, which may indicate prolonged blockade of V_2_ receptor by tolvaptan for >24 h [51]. In contrast, urine aquaporin-2 remained undetectable during tolvaptan treatment in the non-responders [51]. Persistent undetectable level of urine aquaporin-2 regardless of tolvaptan administration indicates lack of response to tolvaptan.

## 4. Conclusions

In conclusion, urine aquaporin-2 is a novel predictor of responsiveness to tolvaptan, and the responders achieved amelioration of symptomatic congestion, normalization of hyponatremia, and improvement in renal function during tolvaptan treatment. Long-term prognosis in the aquaporin-defined responders during tolvaptan treatment should be confirmed in a prospective randomized trial in the future.

## Abbreviations

HF: heart failure
AVP: arginine vasopressin
U-OSM: urine osmolality
